# A partial convolution generative adversarial network for lesion synthesis and enhanced liver tumor segmentation

**DOI:** 10.1002/acm2.13927

**Published:** 2023-02-17

**Authors:** Yingao Liu, Fei Yang, Yidong Yang

**Affiliations:** ^1^ Department of Engineering and Applied Physics University of Science and Technology of China Hefei Anhui China; ^2^ Department of Radiation Oncology University of Miami School of Medicine Miami Florida USA; ^3^ Department of Radiation Oncology the First Affiliated Hospital of USTC Division of Life Sciences and Medicine University of Science and Technology of China Hefei Anhui China; ^4^ School of Physical Sciences & the Ion Medical Research Institute University of Science and Technology of China Hefei Anhui China

**Keywords:** generative adversarial network, lesion synthesis, liver lesion segmentation, mask synthesis

## Abstract

Lesion segmentation is critical for clinicians to accurately stage the disease and determine treatment strategy. Deep learning based automatic segmentation can improve both the segmentation efficiency and accuracy. However, training a robust deep learning segmentation model requires sufficient training examples with sufficient diversity in lesion location and lesion size. This study is to develop a deep learning framework for generation of synthetic lesions with various locations and sizes that can be included in the training dataset to enhance the lesion segmentation performance. The lesion synthesis network is a modified generative adversarial network (GAN). Specifically, we innovated a partial convolution strategy to construct a U‐Net‐like generator. The discriminator is designed using Wasserstein GAN with gradient penalty and spectral normalization. A mask generation method based on principal component analysis (PCA) was developed to model various lesion shapes. The generated masks are then converted into liver lesions through a lesion synthesis network. The lesion synthesis framework was evaluated for lesion textures, and the synthetic lesions were used to train a lesion segmentation network to further validate the effectiveness of the lesion synthesis framework. All the networks are trained and tested on the LITS public dataset. Our experiments demonstrate that the synthetic lesions generated by our approach have very similar distributions for the two parameters, GLCM‐energy and GLCM‐correlation. Including the synthetic lesions in the segmentation network improved the segmentation dice performance from 67.3% to 71.4%. Meanwhile, the precision and sensitivity for lesion segmentation were improved from 74.6% to 76.0% and 66.1% to 70.9%, respectively. The proposed lesion synthesis approach outperforms the other two existing approaches. Including the synthetic lesion data into the training dataset significantly improves the segmentation performance.

## 1 INTRODUCTION

Liver cancer is the fifth most common cancer and the second leading cause of cancer‐related death worldwide.^1^ Accurate diagnosis of liver lesions in CT exams is crucial for the following disease management. Lesion segmentation is an important task in the process of diagnosis and treatment. For example, accurate segmentation is required to clearly define the target volume in the process of radiation therapy treatment planning.

In recent years, deep learning has made significant achievements in medical image segmentation.^2^, ^3^ A large amount of labeled data covering sufficient data diversity, including various lesion locations and lesion sizes, is necessary for the development of a robust deep learning model. On the other hand, manual lesion annotation, a time‐consuming and labor‐intensive task, is usually required to work as the ground truth for segmentation verification. One way to solve both the data paucity and manual labeling problems is to generate synthetic lesions with deep learning networks.

The state‐of‐art deep learning network for lesion synthesis is the generative adversarial network^4^ (GAN). Frid‐Adar et al.^5^ utilized a GAN to generate synthetic images to improve liver lesion classification. The initial GAN usually generates images without any labeling. In the segmentation, however, images with lesion contours are necessary. Isola et al.^6^ introduced conditional GAN (cGAN) which can generate new images with classified data labels. Abhishek et al.^7^ applied the method to synthesize lesions while keeping their original contours and then used the synthesized lesions to augment the training dataset for enhanced lesion segmentation. Likewise, Jin et al.^8^ further developed a 3‐dimensional (3D) cGAN to simulate labeled lung nodules for enhanced lung segmentation.

Despite the promising achievements of GAN, the standard convolution operation in GAN applies same filters to all pixels, both inside and outside the mask, inevitably leading to blurred lesion borders or lesion texture loss. To deal with this problem, partial convolution^9^ is proposed to apply to only pixels outside a mask. Dong et al.^10^ utilized 3D partial convolution to reconstruct the missing regions in ultrasound images using least squares generative adversarial network^11^ (LSGAN) network. Zhang et al.^12^ employed partial convolution to generate synthetic hemorrhage lesions for improved intracranial hemorrhage diagnosis using cGAN network.

Inspired by these methods, we developed a new partial convolution GAN (PCGAN) which can generate synthetic lesions with predefined lesion contours and more realistic textures. Including the synthetic lesions generated from the proposed network into the training dataset can increase the data diversity and therefore enhance the performance of deep learning‐based lesion segmentation.

## 2 MATERIALS AND METHODS

### 2.1 Image dataset

In this study, we used the public dataset from LITS^13^ which contains 131 subjects with liver cancer. We selected lesions larger than 10 pixels for reliable lesion texture assessment,^14^ resulting in 6612 images with lesions from 117 subjects. To accelerate the training process, all the CT images were resized to 256 × 256 pixels from 512 × 512 pixels. The liver in each image was extracted using the contour drawn by physicians. The image window was set to [−100, 200] HU and normalized to [0,1]. In our work, we use two kinds of networks, a lesion texture generation network to generate synthetic lesions and a segmentation network for lesion segmentation. All images were divided into three sets and the training/validation/testing set each contains 3454/1618/1540 images from 87/18/12 patient CT scans. While the data division was same, the training strategies were different. The lesion texture generation network did not use cross‐validation, while the segmentation network was trained in five‐fold cross‐validation to obtain more reliable segmentation results.

### 2.2 Liver lesion synthesis

**FIGURE 1:**
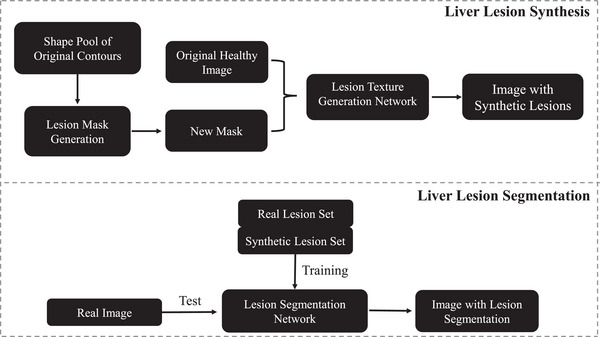
The lesion segmentation workflow. It consists of three stages. The first stage is to automatically generate new masks based on the real tumors in the training dataset. The new masks are additional and they do not affect the existing tumors. The second stage is to produce synthetic lesion textures on healthy liver tissues. The last stage is to segment real lesions using a well‐trained network.

Liver lesions appear in various shapes. The shape of a lesion is an important parameter and should be modeled reasonably by mimicking real ones. The proposed lesion segmentation strategy is described in Figure 1, and the details of the method are described below.

#### 2.2.1 Lesion mask generation

**FIGURE 2:**
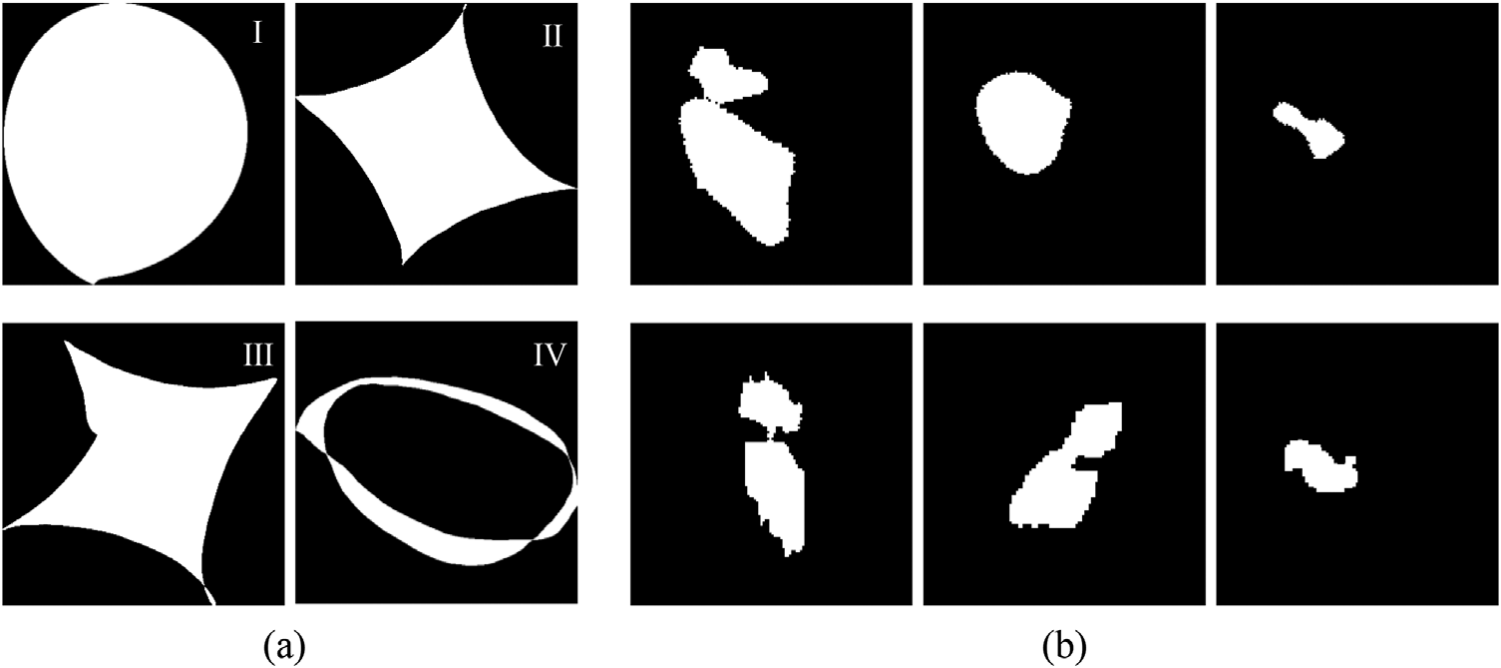
(a) 1st, 2nd, 3rd, and 4th principal mode extracted from the PCA modeling of existing lesion shapes. (b) Some samples of our generated masks (1st row) and original masks (2nd row).

First, we used principal component analysis (PCA) to perform shape decomposition and to analyze the shape variation. All the lesion masks are aligned with each other and then scaled to the same area. Empirically, 200 points equally spaced along the boundary of a mask were extracted to form a vector ${\left[x_{1},\;x_{2},\;\text{…},\;x_{n},\;y_{1},\;y_{2},\;\text{…},\;y_{n}\right]}^{T}$ representing the shape of the mask, where $x_{i}$ and $y_{i}$ are the position of the i^th^ point. Then the Procrustes analysis^15^ is employed to mathematically register all the shapes by taking the rotation and scaling effects into account. Finally, PCA is employed to do dimensionality reduction in the sample dimension. Ten principal components are used, and the first four principal components are demonstrated in Figure 2a. The first component can be interpreted as the shape average of all the masks, while the remaining modes reflect the shape variation at different orders of magnitude. We use $D={\left[D_{1},\;D_{2},\;\text{…},\;D_{10}\right]}^{T}$to denote the first 10 decomposed components and $w\;=\;[w_{1},\;w_{2},\;\text{…},\;w_{10}]$ the corresponding weights. Thus, a new shape can be generated through a transformation operation T(wD) by varying the weight w. Here the purpose of the transformation T( · ) is to diversify the location, size, and rotation of the synthetic lesion. The area of the new shape is not fixed, but follows a distribution as that of the tumors in the training set. Some examples of new masks and original masks are provided in Figure 2b.

#### 2.2.2 Lesion texture generation

**FIGURE 3:**
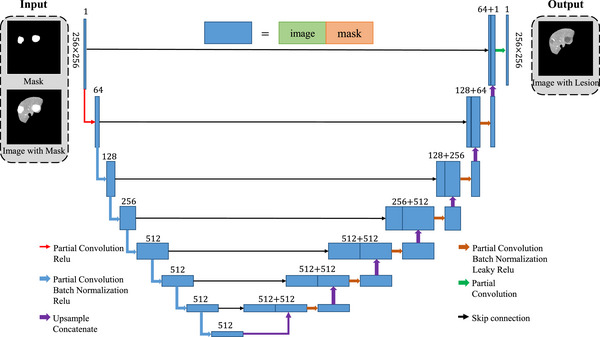
The structure of the U‐Net lesion texture generator. It is built with partial convolutional layers. The input is a binary mask and a masked image. The output is an image with synthetic lesions. The encoder and decoder part each has eight stages. Skip connection is applied to each stage.

The generator, illustrated in Figure 3, is built with partial convolutional layers. The input is a binary mask and an CT image bearing this mask. In the training stage, the mask is obtained from physicians’ lesion delineation. In the testing stage, automatically generated masks are applied. The purpose of using partial convolution is to make the convolution process dependent only on the unmasked pixels via a re‐normalization step. The convolutional result at each location is defined as:

(1) $$x^{\prime }=\left\{\begin{array}{cc} (W^{T}\left(X⊙M\right)\frac{1}{\sum (M)}+b, & \;if\;\sum (M)>0\\ 0, & \;otherwise \end{array}\right.$$

where *W* is the weight of the filter and b is the bias of the filter. ⨀ represents convolution operation. *X* is the feature values for the current convolution window and *M* is the corresponding binary mask. *X* is updated from layer to layer.

Meanwhile, the binary mask is also simultaneously updated with the convolution process via a customized operation. In the operation, a kernel with a size same to that of the convolution window is first defined. If there is any pixel in the mask that has a value of 1, then all the pixels covered by the kernel will be assigned the value 1. After enough updates, all the pixels in the mask will finally have a value of 1, and all the pixels in the image will become unmasked.

The generator is a U‐Net^16^ variant and consists of 8 stages. Relu is used as the activation function in the encoder part, while LeakyRelu with a slope parameter of 0.2 is used in the decoder part. Batch normalization^17^ is adopted for all the convolutional layers except the first and last layers. Skip connection is also introduced to ensure feature reusability by concatenating the feature maps of the encoders and decoders of the same stages.

**FIGURE 4:**
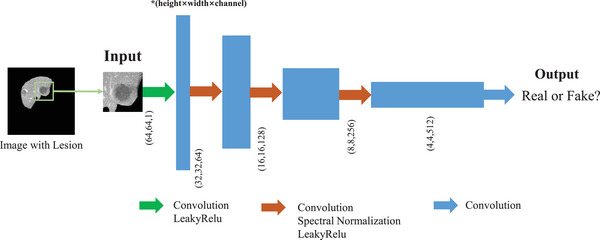
The structure of the discriminator. The input is a 64 × 64 matrix with a randomly‐selected single lesion. There are 4 layers in the discriminator. Each of the first three layers has a kernel size of 4, a stride of 2, and a padding of 1. In the last layer, the output is reduced to one channel and obtain a prediction probability.

The architecture of the discriminator$\;D_{l}$ is illustrated in Figure 4. The input is a 64 × 64 matrix with a randomly‐selected single lesion. There are 4 layers in the discriminator. Each of the first three layers has a kernel size of 4, a stride of 2, and a padding of 1. In the last layer, the output is reduced to one channel and obtain a prediction probability. Spectral normalization^18^ is employed after all intermediate convolutional layers to stabilize the training of the networks.

The generator and the discriminator are trained to minimize a total loss function, which is defined as:

(2) $$\begin{array}{ccc} L_{total} & = & L_{GAN}+\lambda_{reconstruction}L_{reconstruction}\\ & & +\;\lambda_{perceptual}L_{perceptual}+\lambda_{texture}L_{texture} \end{array}$$

$\lambda_{reconstruction}$, $\lambda_{perceptual}$ and $\lambda_{texture}$ are introduced mainly to weigh the importance of the four losses. After optimization, we set $\lambda_{reconstruction}=1$, $\lambda_{perceptual}=0.05$ and $\lambda_{texture}=100$.

$L_{GAN}$ is designed to encourage the generator to generate more similar textures between the generated and real lesions with the supervision of the discriminator. The Wasserstein GAN^19^ (WGAN) loss is chosen as our loss function. The generator and discriminator are trained by solving an adversarial problem:$\;\min_{G}\underset{D}{\;\max\;}L_{GAN}\left(G,D\right)$, and the GAN loss is defined as:

(3) $$L_{GAN}\left(G,\;D\right)=E\left[D\left(x_{i}⊙M_{i}\right)\right]-E\left[D\left({\hat{x}}_{i}⊙M_{i}\right)\right]-\lambda_{gp}G_{p}\left(D\right)$$

where $x_{i}$, $\hat{x_{i}}$, and M_*i*_ represents the i^th^ real image, its corresponding synthetic image and binary mask. $G_{p}\left(D\right)$ is the gradient penalty to enforce the Lipschitz constraint.

Like that in previous studies,^7^, ^8^ $L_{reconstruction}$ is adopted to encourage the decoder to quickly produce a rough outline of the predicted object for better optimization, and is defined as:

(4) $$\begin{array}{ccc} & & L_{reconstruction}-w_{1}{\left\|M_{i}⊙\left(x_{i}-{\hat{x}}_{i}\right)\right\|}_{1}\\ & & \;+\;w_{2}{\left\|\left(1-M_{i}\right)⊙\left(x_{i}-{\hat{x}}_{i}\right)\right\|}_{1} \end{array}$$

where ⨀ denotes element‐wise multiplication. *w*_1_, *w*_2_ are used to weigh the importance of healthy tissue and lesion. After optimization, we set $w_{1}=1$ and $w_{2}=5$.

$L_{perceptual}$ is utilized to capture the high‐level semantics and simulate the human perception of images quality. Perceptual loss^20^ is defined as:

(5) $$\begin{array}{ccc} & & L_{perceptual}\\ & & \;=\;E\left[\sum_{i}\frac{1}{N_{i}}\left({\left\|\Phi_{i}\left({\hat{x}}_{i}\right)-\Phi_{i}\left(x_{i}\right)\right\|}_{1}+{\left\|\Phi_{i}\left(z\right)-\Phi_{i}\left(x_{i}\right)\right\|}_{1}\right)\right] \end{array}$$

where $N_{i}$ denotes the number of pixels in $x_{i}$, $\Phi_{i}$ is the feature map of the i^th^ layer of VGG‐16^21^ network pretrained on ImageNet,^22^ Here, low‐level(the first 5 layers), mid‐level(the 6th layer to 10th layer), and high‐level(the 11th layer to 17th layer) features of VGG network are employed to simultaneously calculate the perceptual and texture losses. z is a composite of the real image and the predicted image, which is defined as $z=M_{i}⊙x_{i}+\left(1-M_{i}\right)⊙{\hat{x}}_{i}$.

$L_{texture}$ has a same form as $L_{perceptual}$. But the former does not act directly on the feature maps as the later does. Instead, L is calculated based on the autocorrelation (Gram matrix)^23^ of all the feature maps. It doesn't consider the individual pixel position but focus on the correlation between pixels. Given any feature map of size $C_{j}\times H_{j}\times W_{j}$, the texture loss is defined as follows:

(6) $$\begin{array}{ccc} & & L_{texture}\\ & & \;=\;E_{j}\left[\sum_{i}\frac{1}{N_{i}}\left(G_{j}^{\Phi }\left(\hat{x_{i}}\right)-G_{j}^{\Phi }{\left(x_{i}\right)}_{1}+G_{j}^{\Phi }\left(z\right)-G_{j}^{\Phi }{\left(x_{i}\right)}_{1}\right)\right] \end{array}$$

where $G_{j}^{\Phi }$ is a $C_{j}\times C_{j}$ Gram matrix constructed from the selected feature maps.

The lesion synthesis network is optimized using AMSGrad^24^ with $\beta_{1}=0.5$ and $\beta_{2}=0.999$. Kernels are initialized using the method described in the reference.^25^ The generator and discriminator are simultaneously updated, and the learning rates are set to 0.0001 and 0.00001, respectively. The training was conducted on an NVIDIA RTX 3090 GPU (24GB) with a batch size of 6, epochs of 800, and took 28 h.

#### 2.2.3 Evaluation of synthetic lesions

Radiomics features (GLCM‐energy and correlation) are utilized to evaluate the realism of synthetic lesions. We computed the feature distributions of synthetic lesions and compared them with real lesions. The similarity between the distributions was assessed by using Kullback–Leibler divergence (KL), which is given as follows:

(7) $$KL(h_{1}\left|\right|h_{2})=\sum_{i=0}^{n-1}h_{1}\left(i\right)\ln\frac{h_{1}\left(i\right)}{h_{2}\left(i\right)}$$

where *n* is the bins of the histogram. *h*_1_ and *h*_2_ are normalized histograms. h(i) denotes the height of the i^th^ bin. KL is to calculate the asymmetry of the difference between two discrete distributions *h*_1_ and *h*_2_.

The synthetic lesions are generated using the proposed PCGAN, and the results are benchmarked against two other typical methods, including cGAN and Tub‐sGAN. As for cGAN, the network architecture reported by Abhishek et al.^7^ was used which performs well in skin lesion synthesis for enhanced lesion segmentation. The Tub‐sGAN^26^ network is the first to incorporate style transfer into the GAN framework. It adopted a cGAN architecture that combined style, content, and L1 losses together. These two networks are trained with the default parameters published in the original study.

### 2.3 Enhanced lesion segmentation

#### 2.3.1 Network architecture

Three widely used semantic segmentation networks: U‐Net, Attention U‐Net,^27^ and U‐Net++^28^ are employed. We adopted the original architecture of these networks. These three networks have the same batch size, epoch, and learning rate in the training process. The batch size is set to 16, the epoch to 150, and the learning rate to 0.0003, respectively. A combination of cross‐entropy loss^29^ and Dice loss^30^ is applied as the loss function, which is defined as:

(8) $$L_{CE-Dice}=\lambda_{CE}L_{CE}+\lambda_{Dice}L_{Dice}$$

(8a) $$\begin{array}{ccc} & & \mathrm{With}\;L_{CE}\left(y,\hat{y}\right)\\ & & \;=\;-\frac{1}{N}\sum_{i}y\log\left(\hat{y}\right)+\left(1-y\right)\log\left(1-\hat{y}\right),L_{Dice}\left(y,\hat{y}\right)\\ & & \;=\;1-\frac{2y\hat{y}+1}{y+\hat{y}+1} \end{array}$$

where $\hat{y}$ refers to the predicted value and *y* to the ground truth label. In Dice loss, 1 is added in both numerator and denominator to ensure that the function is not undefined in boundary scenarios such as when *y* = $\hat{y}$ = 0. $\lambda_{Dice}$ and $\lambda_{CE}\;$are set to 1 and 0.5, respectively.

#### 2.3.2 Evaluation of segmentation

The metrics used to evaluate the segmentation performance include dice similarity coefficient (DSC), volume precision (vPSC), and volume sensitivity (vSEN)

(9) $$\mathrm{DSC}\left(\%\right)=\frac{2\left(V_{pre}\cap V_{gt}\right)}{V_{pre}+V_{gt}}\times \;100\%$$

(10) $$\mathrm{vPSC}\left(\%\right)=\frac{V_{pre}\cap V_{gt}}{V_{pre}}\times \;100\%$$

(11) $$\mathrm{vSEN}\left(\%\right)=\frac{V_{pre}\cap V_{gt}}{V_{gt}}\times \;100\%$$

where $V_{pre}$ and $V_{gt}$ represent the predicted and ground truth lesion volume.

## 3 RESULTS

### 3.1 Evaluation of synthetic lesions

**FIGURE 5:**
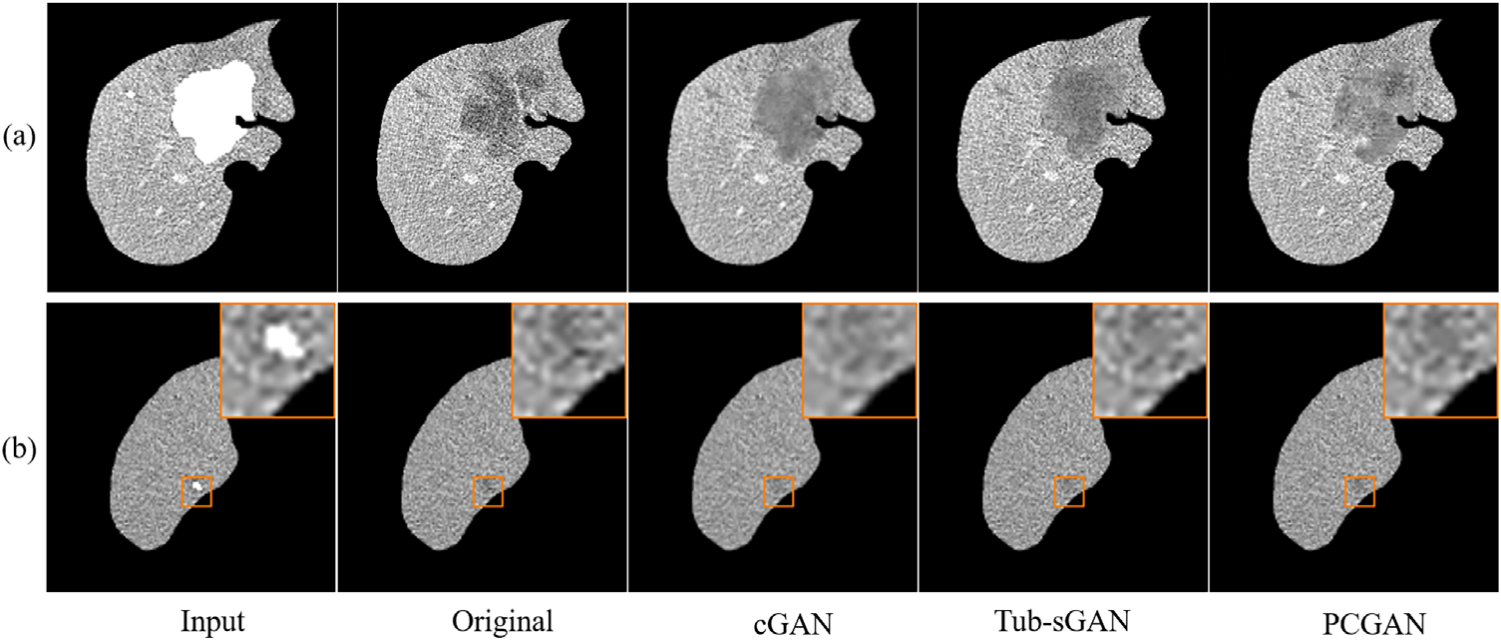
Synthesis results of different lesion synthesis methods using two infected slices and their corresponding manual contoured masks in the test set.

The similarity in the image appearance between the synthetic and real lesions were first evaluated. Figure 5 compares the synthetic lesions generated on real infected image slices using different texture‐generation networks. The original lesion contour manually delineated by physicians was used as the lesion mask. Figure 5a shows the lesion generated by the proposed network PCGAN blend well into the surrounding healthy tissues, and has more heterogeneous texture than those generated with cGAN and Tub‐sGAN. Figure 5b gives an example of a small lesion about 2‐mm in dimension. The lesion boundary is almost lost for the cGAN but is well maintained for the PCGAN.

**FIGURE 6:**
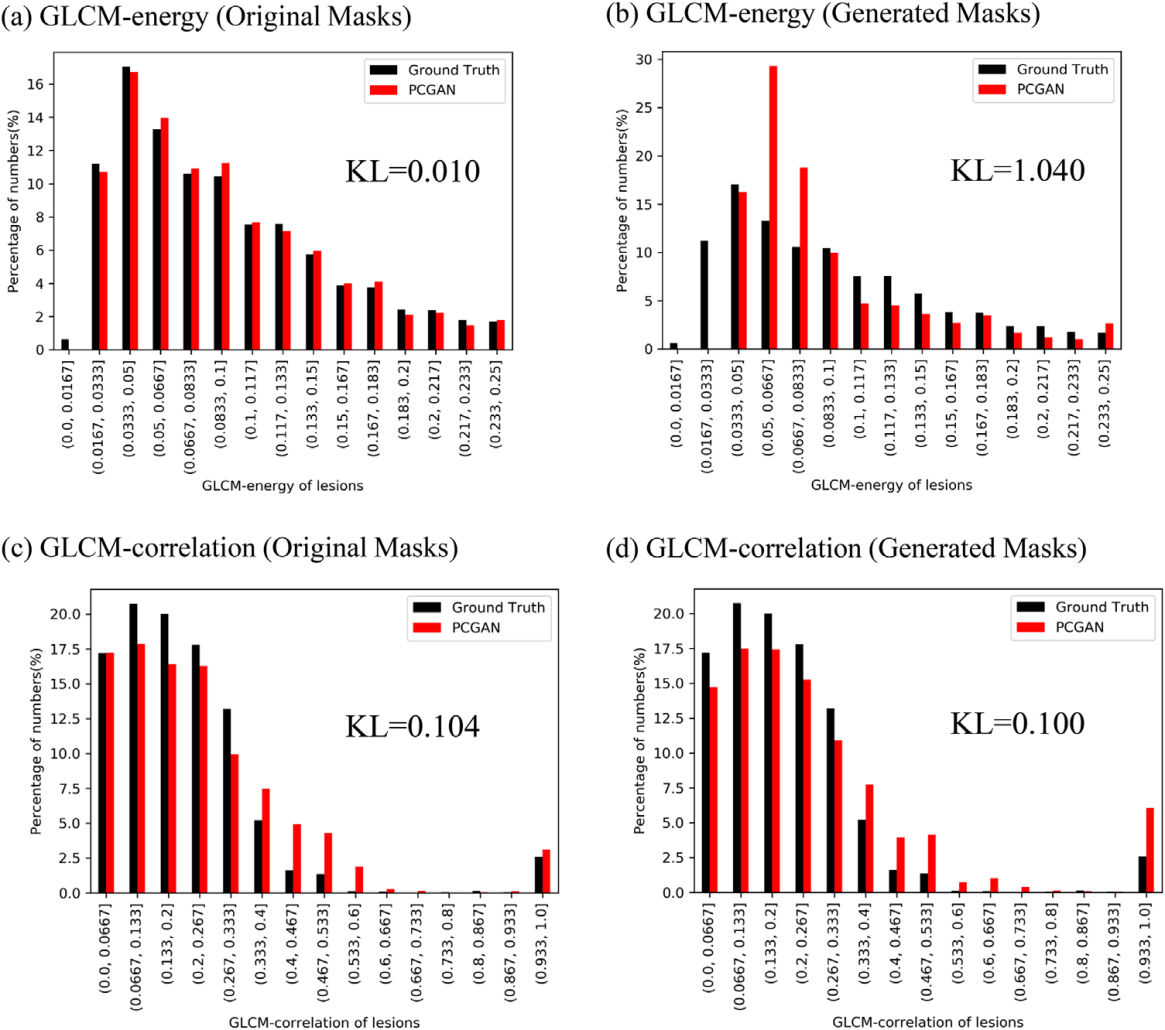
The radiomics features histograms for real images and synthetic images generated by partial convolution GAN (PCGAN). Kullback–Leibler divergence (KL) is applied to compute the distances of two radiomics features distributions.

Two commonly used radiomics features, GLCM‐energy and GLCM‐correlation, were applied to compare the collective similarity between the synthetic and real lesion set. As shown in Figure 6, the synthetic and real lesion set have very similar distributions for the two parameters GLCM‐energy and GLCM‐correlation, particularly for those generated using the original contours. To make this figure more readable, the radiomics features histograms of other methods are also given in the Supplementary Material. For GLCM‐energy, PCGAN has the KL score of 0.010 and performs best in these three methods. For GLCM‐correlation, Tub‐sGAN and PCGAN perform better, with their KL scores of 0.079 and 0.104, respectively. The GLCM‐correlation distributions for those created with generated masks still achieve low KL scores, and PCGAN has the lowest KL score of 0.100. However, the GLCM‐energy distributions for those created with generated masks are slightly off from the ground truth. The KL scores for cGAN, Tub‐sGAN, and PCGAN are 1.239, 1.183, and 1.040, respectively.

### 3.2 Evaluation of enhanced lesion segmentation

**FIGURE 7:**
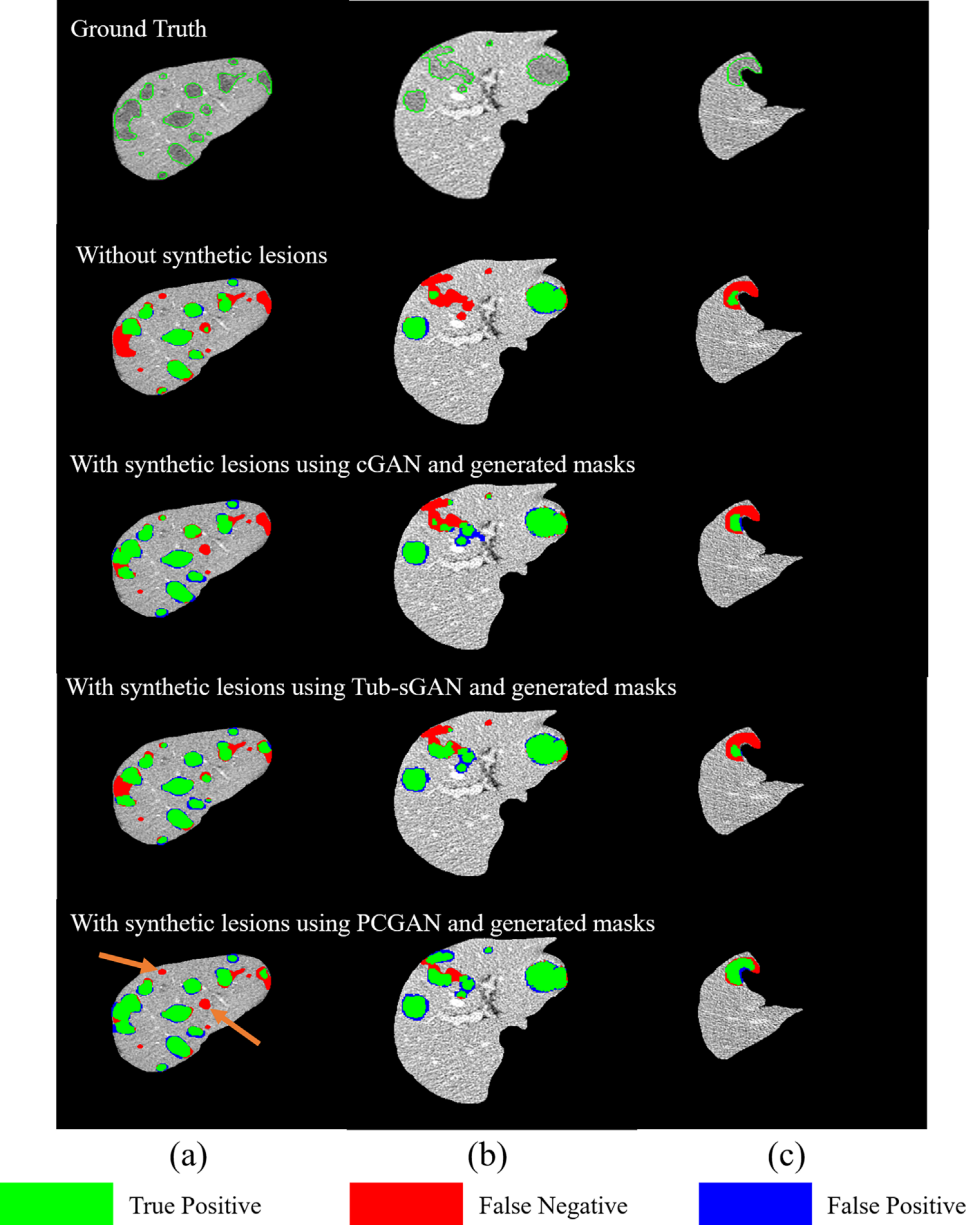
Comparison of enhanced lesion segmentation using networks trained with different lesion synthesis methods. (a–c) show segmentation results from different patients. The green, red, and blue areas are true positive, false negative, and false positive, respectively.

Figure 7 demonstrates the lesion segmentation performance when including the synthetic lesions in the training data set. The lesion segmentation network was a classic U‐Net which is heavily used in segmentation tasks. The demonstrated three lesions are varied in the location, size, and shape. Interestingly, Figures 7a gives an extreme case with 15 separate lesions. The proposed network PCGAN produced both less false positives and less false negatives. However, it still misses two small lesions, as indicated by the orange arrows in Figure 7a. In Figure 7b and c, PCGAN delineates more accurate lesion boundaries. Particularly for the small lesion with an irregular shape, PCGAN segmented almost all the lesion volume while the other two networks missed most of it. The segmentation performance on the testing dataset is given in Table 1. The segmentation results are obtained by training a U‐Net/Attention U‐Net/U‐Net++ using the real training data and synthetic images generated by different lesion synthesis methods. As shown in Table 1, when the training dataset is augmented with the synthetic images generated by different synthesis methods, the segmentation performance all improves significantly. The averaged results reach a DSC difference of 2.7% and 4.5% in U‐Net and Attention U‐Net, and both are significant (*p* < 0.05). In U‐Net++, the DSC difference is 1.8% and is not significant. The differences of vPSC and vSEN are 1.1%, 2.8% in U‐Net, 2.7%, 3.3% in Attention U‐Net and 2.3%, 0.8% in U‐Net++.

**TABLE 1:** Segmentation performance using lesions synthesized with different methods

Methods		DSC(%)	vPSC(%)	vSEN(%)
U‐Net	Real	67.3±19.0	74.6±21.6	66.1±17.0
	Real+Syn[Averaged]	70.0±17.2	75.7±20.4	68.9±15.5
	Real+Syn[cGAN]	69.1±18.1	74.6±21.5	68.9±15.0
	Real+Syn[Tub‐sGAN]	69.4±17.2	76.5±20.0	66.8±15.6
	Real+Syn[PCGAN]	71.4±16.2	76.0±19.5	70.9±14.7
Attention U‐Net	Real	64.9±22.0	71.0±24.3	66.7±17.6
	Real+Syn[Averaged]	69.4±18.7	73.7±21.8	70.0±15.1
	Real+Syn[cGAN]	68.6±20.0	71.6±23.6	70.9±14.7
	Real+Syn[Tub‐sGAN]	69.2±18.3	75.0±21.0	68.1±15.9
	Real+Syn[PCGAN]	70.3±17.7	74.6±20.5	71.0±14.6
U‐Net++	Real	68.0±18.8	75.0±20.8	66.8±18.0
	Real+Syn[Averaged]	69.8±17.4	77.3±20.0	67.6±16.8
	Real+Syn[cGAN]	70.1±17.2	76.2±20.8	69.1±15.2
	Real+Syn[Tub‐sGAN]	67.1±18.9	79.1±20.2	62.2±19.1
	Real+Syn[PCGAN]	72.1±15.7	76.7±18.8	71.4±14.2

*Notes*: The best results are highlighted in bold. “Real” and “Syn” refer to 3454 real images and 1731 synthetic images. Both real images and synthetic images are used to train segmentation networks. “Averaged” is the average result.

Abbreviations: DSC, dice similarity coefficient; vPSC, volume precision, vSEN, volume sensitivity.

Compared with other lesion synthesis methods, the proposed PCGAN results in higher segmentation scores in DSC and vSEN. The differences of DSC are 4.1% in U‐Net, 5.4% in Attention U‐Net and 4.1% in U‐Net++, and are all significant (*p* < 0.05). The differences of vPSC and vSEN are 1.4%, 4.8% in U‐Net, 3.6%, 4.3% in Attention U‐Net and 1.7%, 4.6% in U‐Net++.

## 4 DISCUSSION

In this study, we propose a lesion synthesis method to generate labeled training image samples for enhanced lesion segmentation. When the training dataset is augmented with the synthetic images, all the segmentation networks under evaluation achieved improved performance, due to the increased lesion diversity in the training dataset brought in by the synthetic lesions. Compared with other lesion synthesis methods, cGAN and Tub‐sGAN, the proposed PCGAN can generate lesions with more realistic textures.

There are still some limitations in the proposed method. First, the texture evaluation needs further investigation. The current work focuses on using GLCM features for texture evaluation because it represents pixel and spatial relationships well. Previously, Pan et al.^14^ evaluated the synthetic lung lesions using GLCM homogeneity, contrast, and energy. In our work, GLCM‐energy and GLCM‐correlation are used to evaluate the texture of synthetic liver lesions. However, GLCM features are not invariant to rotation changes in the texture because they are computed in a specified direction. To compensate for this problem, future work may consider using more radiomics features, such as GLSZM, GLRLM, and GLDM.^31^

On the other hand, the network was trained with 2D images due to hardware limitations and data scarcity. This strategy didn't make full use of the three‐dimensional property of CT images. Future studies using 3D image input may improve synthesis quality, particularly in terms of the lesion texture transition along the image thickness direction. In addition, only one constraint parameter of lesions, shape information, is introduced to supervise the synthesis network. In the future, more constraint parameters, such as density, energy, and other radiomics features, can be used to finetune the appearance of synthetic lesions.

## 5 CONCLUSION

In this paper, we present a novel lesion synthesis approach to generate labeled training image samples for enhanced lesion segmentation. The synthetic lesions are generated on healthy image slices using automatically generated contours on a new PCGAN. The segmentation performance is significantly improved after the training dataset is augmented with the synthetic images. The approach shows great potential to alleviate the “data paucity” problem in image‐based lesion segmentation.

## AUTHOR CONTRIBUTIONS

*Study design, data analysis, and manuscript drafting*: Yingao Liu. *Manuscript revision*: Fei Yang. *Study guidance, manuscript revision, and financial support*: Yidong Yang.

## CONFLICT OF INTEREST STATEMENT

The authors have no conflict of interest to disclose.

## Supporting information

SUPPORTING INFORMATION
