# Supplementary material for: A partial convolution generative adversarial network for lesion synthesis and enhanced liver tumor segmentation
Source: J Appl Clin Med Phys. 2023 Feb 17;24(4):e13927. doi: 10.1002/acm2.13927 (PMC10113707; doi:10.1002/acm2.13927)
Supplement: Supplementary file 1 — SUPPORTING INFORMATION [file ACM2-24-e13927-s001.docx]

In this supplementary material, radiomics features histograms of cGAN^7^ and Tubs-GAN^23^ is provided.


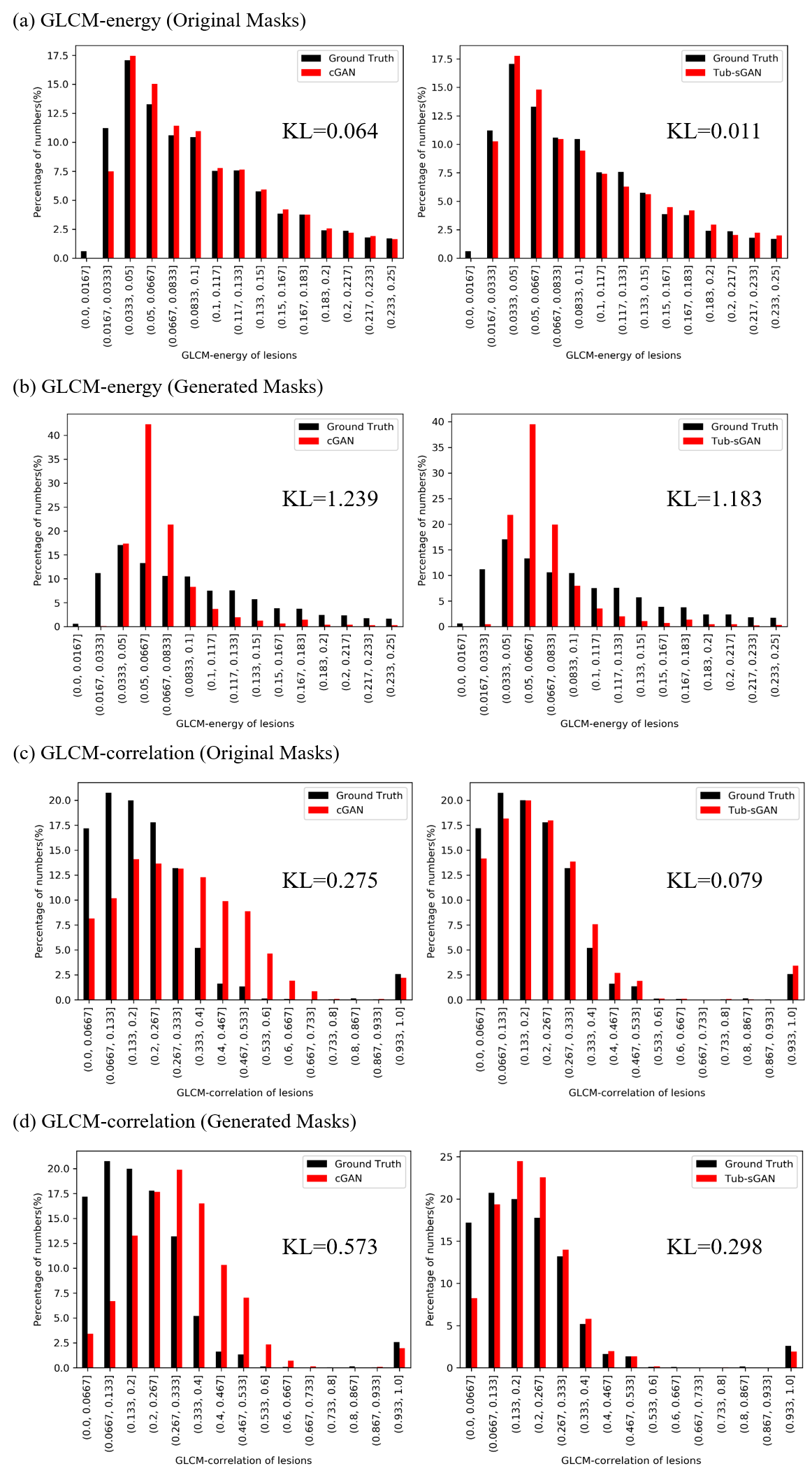


Figure 8: The radiomics features histograms for real images and synthetic images generated by cGAN and Tub-sGAN. Kullback-Leibler divergence (KL) is applied to compute the distances of two radiomics features distributions.
